# Medical genetics and genomic medicine in the United States. Part 2: Reproductive genetics, newborn screening, genetic counseling, training, and registries

**DOI:** 10.1002/mgg3.343

**Published:** 2017-11-26

**Authors:** Debra S. Regier, Carlos R. Ferreira, Suzanne Hart, Donald W. Hadley, Maximilian Muenke

**Affiliations:** ^1^ Rare Disease Institute Children's National Health System Washington DC USA; ^2^ National Human Genome Research Institute National Institutes of Health Bethesda MD USA

## Abstract

Review of genetics in the United States with emphasis on the prenatal, metabolic, genetic counseling, and training aspects of the field.

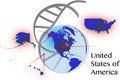

## 1 INTRODUCTION

Modern Americans cannot live a day without being inundated by genetic and genomic information. From television commercials promoting DNA‐based testing for colon cancer and ancestry panels to Internet ads for DNA testing for drug interactions and to find the perfect life partner, DNA‐based testing is expected to cure, entertain, and even find all of us happiness. As the first Genetics in the USA article showed the long history of genetics in creating a culture of diagnosis and care for patients, this article will continue the story and share the advances in the last century and how modern medicine, everyday life, and our every day beliefs have been affected by genetics. Or in short, how DNA has “helixed” itself into the culture of the United States.

## 2 THE HISTORY OF DNA

Europe was the home of early DNA understanding. In the 1860s, the Austrian Gregor Mendel described his experiments in peas showing inheritance and became known as the father of genetics. In the 1950s the structure of DNA was elucidated with the work of Rosalind Franklin, Maurice Wilkins, Francis Crick, and the American James Watson while in Europe. In the 1970s a group in Europe developed Sanger sequencing (Sanger, Nicklen, & Coulson, 1977), whereas the Massachusetts based group of Maxam and Gilbert developed a chemical sequencing mechanism (Maxam & Gilbert, 1977). While Sanger sequencing went on to become the more cost‐effective mechanism, this showed the cooperation between scientists in Europe and the United States to move forward the field of genetics. In 1983 Kary Mullis described the polymerase chain reaction, or PCR, which allowed for DNA to be efficiently copied in the laboratory (Mullis et al., 1986). His work to identify thermostable polymerases earned him the Nobel Prize in Chemistry in 1993. It was the combination of Sanger sequencing, computational abilities made possible by super computers, and PCR that allowed for the launch of the Human Genome Project, an international scientific project to determine the sequence of base pairs that makes up the human genome with the goal of creating a physical and functional map. This project was launched in 1990 and funded by the National Institutes of Health and the Department of Energy. The project included groups from across the United States and the world. Its completion in April 2003 was 2 years ahead of schedule. By then, over 92% of the human genome had been sequenced, which included over 99% of euchoromatic DNA, with greater than 99.99% accuracy (Schmutz et al., 2004). The key findings of this multiyear, multinational project were the following:

1. 22,300 protein‐coding genes in humans, similar to other mammals (Pertea & Salzberg, 2010);
2. Larger numbers of segmental duplications than had been previous expected (International Human Genome Sequencing Consortium, 2004);
3. Less than 7% of the protein family groups were vertebrate‐specific.

Since the completion of the human genome project, the project has already led to the discovery of more than 1,800 disease genes, 350 biotechnology‐based products, and 2,000 genetic tests for human conditions (NIH Fact Sheets – Human Genome Project). Now in the post‐Human Genome Project era, the work has shifted to combining the disorders described by earlier clinicians with the DNA information provided. Thus, the post‐Human Genome Project field of medical genetics is a bold new world with more complexities than we ever imagined and more hope for future treatments and improved outcomes than we could have dreamed.

## 3 PRENATAL DIAGNOSIS AND SCREENING

With the delineation of genetic syndromes and the identification of proteins involved, the use of preconception screening became common. First widely used in the Ashkenazi Jewish population for Tay–Sachs disease in the 1970s, by measuring enzyme activity in adults, carriers of the disorder could be detected and reproductive risk addressed in this autosomal recessive disorder. With the expansion of genetic evaluation and sequence changes that are commonly found in rare diseases, screening panels for carrier states became available. Both the American College of Medical Genetics and Genomics (ACMGG) and the American College of Obstetrics and Gynecology (ACOG) have recommended carrier screening in populations at high risk for rare disorders (i.e., Ashkenazi Jews, or in all populations for cystic fibrosis) (Gross, Pletcher, & Monaghan, 2008). From single disorder panels for common variants in the Mennonite, Amish, and Jewish populations to identification of the triplet repeat number that is responsible for Fragile X to a pan‐ethnic screen that covers thousands of the most common genetic changes leading to rare disorders, the use of DNA to identify carriers of rare autosomal recessive, X‐linked, and autosomal dominant disorders has become common place in Western medicine. While the ACMGG and the ACOG have yet to endorse specific broadened panels, they have endorsed ensuring that laboratories performing testing use best practices (Grody et al., 2013). This publication was to ensure accuracy for the tests that are in use and leading to decision‐making either before or during a possibly affected pregnancy.

The FDA has become involved in the field of carrier testing. In November 2013, they warned a direct‐to‐consumer company that their product required FDA approval due to it being a new “medical device” (Warning Letters – 23andMe, Inc. 11/22/13). Over the past several years, the field has evolved to include ancestry testing and commercials asking the consumer to “ask your doctor” for genetic testing. As of the time of writing this article, direct‐to‐consumer testing for ancestry determination is commercially available. However, testing for medically actionable results remain under the regulation of physician orders.

With the ability to leverage DNA information for health advances, the role of this information in prenatal screening and testing has been an ongoing ethical and medical debate. For more than 30 years, prenatal genetics focused on the creation of screening programs that would allow for noninvasive mechanisms to determine risk for fetal disorders. The combination of maternal serum and ultrasound evaluation were the basis of these screens. The emphasis was on Down syndrome, based on the frequency of this disorder. In addition, maternal serum screening was found to be useful for the detection of Down syndrome, trisomy 18, trisomy 13, and neural tube defects. While these screening tests were helpful, the use of chorionic villi sampling—removal of a small piece of placenta—or amniocentesis—removal of a small amount of amniotic fluid—was needed to perform a karyotype, the gold standard test for aneuploidy disorders. For neural tube defects, the second line testing was ultrasound evaluation, as elegantly reviewed by Van den Veyver (2016). With the launch of microarray analysis, a technique that evaluates for submicroscopic deletions and duplications, the use of invasive testing for diagnosis began. In 2013, the ACOG released a recommendation that “in patients with a fetus with one or more major structural abnormalities identified on ultrasonographic examination and who are undergoing invasive prenatal diagnosis, chromosomal microarray analysis is recommended” (American College of Obstetricians and Gynecologists Committee on Genetics, 2013).

The field of prenatal genetic testing had historical acceleration when Lo et al. discovered that fetal DNA could be amplified form maternal plasma (1997). This seminal work launched into clinical significance in 2008 when two groups showed that this fetal DNA could be used to determine fetal aneuploidy status (Chiu et al., 2008; Fan, Blumenfeld, Chitkara, Hudgins, & Quake, 2008). And that was the start of a whole new realm of screening. With sensitivities greater than 98% and specificities in the 90%s, the ACOG now recommends cell‐free fetal DNA evaluation for all women of advanced maternal age. In many clinical practices, this is offered to all patients due to the noninvasive nature of the test. And the ACMGG updated their practice guidelines to recommend “informing all pregnant women that NIPS (noninvasive prenatal screening) is the most sensitive screening option for traditionally screened aneuploidies” (Gregg et al., 2016). In this same publication, they did NOT recommend screening for smaller deletion syndromes due to the sensitivity and specificity of testing (i.e. 22q11 deletion syndrome).

## 4 THE HISTORY OF METABOLIC GENETICS AND NEWBORN SCREENING

In the pregenomics era, biochemical genetics was not dependent on clinical findings alone, but was based on biochemical markers of disease. As DNA‐based genetics has evolved in the last half‐century, so too has the world of biochemical genetics. The earliest identified metabolic disorder, alkaptonuria, was described by British physician Archibald Garrod (Piro, Tagarelli, Lagonia, Quattrone, & Tagarelli, 2010). As certain biochemical markers were identified, the physicians worked to find ways to decrease these levels, in an attempt to create treatment plans. One of the illustrious examples is in the work of Norwegian‐born Asbjørn Følling in the 1930s. He carefully evaluated and identified accumulation of phenylpyruvic acid in the urine of children affected with a disease that thus came to be called phenylketonuria (PKU). In the 1950s, the German physician Horst Bickel identified a low phenylalanine diet as prevention for the neurodisability seen in untreated PKU. This began a new era, where early diagnosis led to a treatment that could prevent or slow down disease progression. When American‐born Robert Guthrie had a niece diagnosed with PKU, he used his skills in bacterial inhibition assays to identify a way to identify patients presymptomatically. He developed an assay that used whole blood that was spotted onto a filter paper card. The Guthrie test and Guthrie card became the first steps in a newborn screening system. In this system, *Bacillus subtilis* was plated on an agar plate with a phenylalanine antagonist. Samples with very high amounts of phenylalanine (i.e., babies affected with PKU) could overcome this inhibition and had rapid growth of the bacteria (Gonzalez & Willis, 2009). By identifying babies with elevated phenylalanine levels BEFORE neurological symptoms occurred, treatment could be begun to prevent complications. This was the launch of the newborn screening program in the United States. Massachusetts was the first state to perform universal, mandatory screening for PKU in 1963. Gradually, the other states adopted PKU screening. Since each state chose the screening to be performed, by the mid 2000s, there was significant disparity between states. The Maternal and Child Health Bureau of the Health Resources and Services Administration (HRSA) and the ACMGG began a joint project to create standardization for states. The 2007 federal Newborn Screening Saves Lives Act of 2007 had provisions to establish seven HRSA‐supported regional centers and support for newborn screening, including the creation of the Recommended Uniform Screening Panel (RUSP). The RUSP is a list of recommended screens from the Secretary of Health and Human Services. The committee that makes these recommendations uses the criteria for disorders that have improved outcomes if identified and treated early (reviewed by Sue Berry, 2015).

Initially, newborn screening was a “one test for one disorder” system, such as that developed for PKU. However, with advances in metabolic medicine, the process of using dried blood spots for evaluation by tandem mass spectrometry allowed for one blood spot to be used to assess for dozens of molecules and conditions. Similarly, using hemoglobin electrophoresis, one sample was able to assess for multiple hemoglobiniopathies simultaneously. This work to increase the effectiveness of the test and decrease the turnaround time on samples has led to a robust and ever‐improving system designed to keep babies safe.

The Centers for Disease Control has included newborn screening on the top ten accomplishments of public health. This program screens more than 90% of babies born in the United States for dozens of disorders that can be treated early to prevent or minimize disease progression. As one of the proudest accomplishments of metabolic genetics, the field of newborn screening is ever advancing. In the last years, the RUSP has continued to recommend screening new disorders, now including some lysosomal storage disorders and peroxisomal disorders.

With the advancements in metabolic diagnosis, the role of improving management has also evolved. Enzyme replacement therapies for lysosomal storage disorders, ammonia scavenger agents for urea cycle disorders, and small molecule cofactors have revolutionized the world of metabolic medicine in the last decades. These treatments along with improved early death prevention have led to a new generation of patients with chronic disease. For example, women with PKU are at a high risk of having babies with major malformations if their phenylalanine levels are not well controlled. While a hundred years ago, this would have never been a public health concern, now the recommendation of “low phenylalanine diet for life” is partly based on these findings (Regier & Greene, 1993).

As the population of children with treated metabolic disorders age, the field continues to discover how the newly treated disorders have their own unique natural histories. The world of treated disorders is only beginning and will certainly be the story of the 21st century in biochemical genetics.

The worlds of biochemical and medical genetics have merged in the realm of prenatal diagnosis. While the gold standard of biochemical genetics is chemical marker testing, identifying patients based on genetic changes has allowed for prenatal diagnosis, identification of groups of patients that will be most amenable to types of treatments, and recurrence risk. Prenatal diagnosis can be performed at various stages. A child with a metabolic disorder can have genetic testing to identify the gene changes leading to this diagnosis. While not always informative, this testing is considered standard of care for many diagnoses. This testing can be used for selection of embryos for implantation in *in vitro* fertilization therapy, for identification of affected fetuses using CVS or amniocentesis, or for early postnatal testing using cord blood or neonatal specimens. Second, genetic sequencing can be used to identify the most optimal treatment plans. For example, the biopku.org database contains hundreds of genetic changes associated with PKU. In each case, they have data showing the response rate to a cofactor treatment that is effective in some patients with PKU (sapropterin dihydrochloride, an isomer of tetrahydrobiopterin). The current guidelines suggest that all PKU patients should have a trial with the cofactor, except those with two truncating mutations in trans (Vockley et al., 2014). The next decades will continue to show how the culture of genetics will create precision medicine for many diseases, metabolic, and chromosomal.

## 5 TRAINING IN MEDICAL GENETICS AND GENOMICS

The first department of Medical Genetics in the United States was established at Bowman Gray School of Medicine (now called Wake Forest University Baptist Medical Center) in 1941. Dr. William Allan was the first chairman and served in that capacity until his death in 1943. The creation of the department was funded by a $50,000 grant from the Carnegie Corporation of New York (Meads & Davis, 1988). Dr. Allan also established the first human genetics course in a medical school. Dr. Allan's records are housed in the Dorothy Carpenter Medical Archives in Winston‐Salem, NC (William Allan Papers, Special Collections at Belk Library). A subset of the records on over 250 families from western North Carolina is currently housed at Appalachian State University. Dr. Allan's contributions to the field of human genetics is recognized by the American Society of Human Genetics (ASHG) through its Allan Award, created in 1961 and awarded “to recognize substantial and far‐reaching scientific contributions to human genetics carried out over a sustained period of scientific inquiry and productivity” (ASHG Awards). ASHG considers the Allan Award to be its highest award.

There are 45 programs in the U.S. that offer Medical Genetics and Genomics (MGG) residencies and fellowships for individuals with an M.D. or D.O (http://www.acgme.org/). These programs are a minimum of 2 years but often require a third year. Combined residencies exist for Pediatrics and MGG (*n* = 16) (List of accredited combined residency programs—MGG‐Peds) and Internal Medicine and MGG (*n* = 5) (List of accredited combined residency programs—MGG‐IM). There are also combined residencies and fellowship programs for Maternal Fetal Medicine and MGG (*n* = 10) (List of accredited combined residency programs—MFM‐MGG) and Reproductive Endocrinology and Infertility and MGG (*n* = 3) (List of accredited combined residency programs—REI‐MGG). Sixteen programs offer training in Medical Biochemical Genetics, a 1‐year program available to individuals who have completed another residency. Molecular Genetic Pathology programs are offered by 39 institutions (http://www.acgme.org/).

Clinicians and PhDs can participate in the laboratory‐based fellowships. Historically, training was offered in Clinical Biochemical Genetics (*n* = 43), Clinical Cytogenetics and Genomics (*n* = 24), and Clinical Molecular Genetics and Genomics (*n* = 42) (ABMGG Accredited Programs—LGG). The laboratory‐based fellowships require 2 years of training, unless another genetics specialty has been completed. In that case the additional specialty can be completed in 1 year. As of July 1, 2017, Clinical Cytogenetics and Genomics and Molecular Genetics and Genomics have been combined into the 2‐year Laboratory Genetics and Genomics (LGG) program (*n* = 29). Some LGG programs require 3 years.

Three groups oversee the training and practice of medical genetics in the United States. Accreditation of medical genetics and genomics residency programs is through the Accreditation Council for Graduate Medical Education (ACMGE; http://www.acgme.org/) Residency Review Committee (RRC). This organization also accredits the Medical Biochemical Genetics and Molecular Genetic Pathology subspecialty programs. The second organization is the American Board of Medical Genetics and Genomics (ABMGG; www.abmgg.org), which accredits the laboratory fellowship programs. Individuals who received their clinical training or PhDs outside of the United States may be eligible to participate in the laboratory fellowships. To determine eligibility, individuals must submit their credentials to ABMGG for review. ACGME recently agreed to assume the accreditation of the laboratory programs. This transition is expected to take approximately 2–3 years. ABMGG also administers the genetics and genomics certification examinations, which are offered in August of odd years. All trainees take an examination of general genetics and genomics knowledge as well as an examination of knowledge specific to the specialty of training. To become certified, the trainee must pass both exams. As of the 2015 examination, there are 1,594 clinical geneticists, 333 clinical biochemical geneticists, 770 clinical cytogeneticists, 685 clinical molecular, 49 clinical biochemical and molecular (examination only offered 1 year), and 63 medical biochemical geneticists (Number of ABMGG‐certified specialists in Medical Genetics and Genomics). Data from the 2017 examination were not available at the time this article was written. The third organization is the American College of Medical Genetics and Genomics (ACMGG; www.acmg.net). ACMGG issues policy statements and practice guidelines for both clinical and laboratory practices, provides educational resources, and works to ensure genetics and genomics are incorporated appropriately into medical practice. The organization also hosts an annual meeting in the spring.

## 6 GENETIC COUNSELING

The term *“*genetic counseling*”* was coined by Sheldon Reed in 1947 (Reed, 1974). Reed, who held a doctorate in Genetics, proposed that the term genetic counseling be used in place of terms to which he objected, for example, genetic consultation, genetic advice (Resta, 2006). The currently accepted definition of genetic counseling was proposed by a committee of the National Society of Genetic Counseling (NSGC) in 2006 and is as follows (National Society of Genetic Counselors’ Definition Task Force et al., 2006):

“The process of helping people was to understand and adapt to the medical, psychological and familial implications of genetic contributions to disease. This process integrates the following:

1. Interpretation of family and medical histories to assess the chance of disease occurrence or recurrence.
2. Education about inheritance, testing, management, prevention, resources, and research.
3. Counseling to promote informed choices and adaptation to the risk or condition.”

While professionals with various backgrounds and training may provide genetic information to patients and their families, certification as a genetic counselor requires specialized training in medical genetics and counseling. Training and certification as a genetic counselor within the United States requires: (1) the successful completion of a Masters level program accredited by the Accreditation Council for Genetic Counseling (ACGC; http://www.gceducation.org) and (2) passing a certifying examination overseen by the American Board of Genetic Counseling (ABGC; http://www.abgc.net/home//). At the time of the submission of this manuscript, there were 37 ACGC‐accredited programs within the United States and 5 additional programs that have submitted a letter of intent proposing new programs. Likewise, there are currently over 4,000 ABGC‐certified genetic counselors within the US.

Since the first genetic counseling program was initiated at Sarah Lawrence College in 1969, the profession has grown dramatically. The U.S. Bureau of Labor Statistics projects a growth rate of 29% for genetic counseling positions over the years from 2014 to 2024. This far exceeds the average growth rate of 7% for all occupations (Genetic Counselors—Occupational Outlook Handbook, U.S. Bureau of Labor Statistics).

The National Society of Genetic Counselors conducts an annual survey of genetic counselors to collect data about the profession (National Society of Genetic Counselors—2016 Professional Status Survey Reports). In 2016, 58% of those responding reported that they work in a “clinical” position, 22% work in a nonclinical position, and 19% report working in a mixed (clinical and nonclinical) position. Of genetic counselors seeing patients, the majority work at a university medical center (36%), public (21%), or private (19%) hospitals with 48% providing services in cancer genetics clinics, 43% in prenatal diagnostic clinics, 25% in pediatrics, 19% in general genetics, 15% adult genetics clinics including complex diseases, and 10% in cardiology clinics. Genetic counselors working in nonclinical positions work primarily in commercial, nonacademic diagnostic laboratories (49%), university medical centers (14%), commercial academic labs (8%), public/private medical facilities (7%), and 22% in a collection of other settings.

Other health care professionals providing genetic counseling services may include clinical Geneticists (physicians), and other genetics subspecialists (biochemical, molecular, and cytogeneticists), genetics nurses, and nongeneticists (primary or specialty care physicians who are not geneticists). There are a significant number of nurses working in Genetics, such that they have their own professional society, the International Society of Nurses in Genetics (ISONG). The genetics nursing credentialing commission uses a portfolio‐based mechanism for appropriately prepared nurses to become credentialed in genetic nursing (http://www.isong.org).

## 7 PATIENT AND PARENT SUPPORT GROUPS, PATIENT ADVOCACY AND PATIENT REGISTRIES

Given the breadth and depth of the topics presented within this section, our intent is to provide a listing of the most widely recognized resources providing an entry point into exploring each topic in greater detail.

### 7.1 Patient advocacy and support resources

Educational and support groups created and maintained by patients and parents fill an enormous void in helping families learn about, cope with, identify resources, and move forward following a diagnosis or the journey to find one. Thousands of patient and family support groups exist and are readily identified through Internet searches. However, we list a few resources that can assist families and health care professionals quickly and safely find credible information and resources to advocate for services on their behalf (Table 1).

**Table 1:** Genetics Resources. The name of the resource, website, and short description of resources are shown that can be helpful for the clinician caring for patients with genetic syndromes

Genetics organization (website)	Short description
The Genetic and Rare Disease Information Center (GARD) https://rarediseases.info.nih.gov/	GARD is a program of the National Center for Advancing Translational Sciences (NCATS) and funded by two parts of the National Institutes of Health (NIH): NCATS and the National Human Genome Research Institute (NHGRI). GARD provides the public with access to current, reliable, and easy to understand information about rare or genetic diseases. Information is available in English or Spanish
Genetics Home Reference (GHR) https://ghr.nlm.nih.gov/	Genetics Home Reference provides consumer‐friendly information about the effects of genetic variation on human health. GHR provides information on: (1) over 1,200 health conditions, diseases and syndromes; (2) over 1,400 genes and the health effects of genetic changes; (3) chromosomes and mitochondrial DNA; and (4) an introduction to fundamental topics related to human genetics, including illustrations and basic explanations of genetics concepts
Genes and Disease, National Center for Biotechnology Information, National Library of Medicine https://www.ncbi.nlm.nih.gov/books/NBK22183/	Collection of articles that discuss genes and the diseases that they cause. These genetic disorders are organized by the parts of the body that they affect. As some diseases affect various body systems, they appear in more than one chapter. With each genetic disorder, the underlying mutation(s) is discussed, along with clinical features and links to key websites
World Health Organization (WHO) http://www.who.int/genomics/en	WHO's Human Genomics in Global Health Initiative aims to provide information and raise awareness within the health sector, governments and the wider public on the health challenges and opportunities within the rapidly developing science of human genomics
The Genetic Alliance www.geneticalliance.org	A nonprofit health advocacy organization that transforms health through genetics, promoting an environment of openness centered on the health of individuals, families, and communities. The Alliance provides a search tool (Disease InfoSearch: http://www.diseaseinfosearch.org/) that will locate quality information from a database of more than 13,000 conditions and thousands of support groups and foundations
The National Organization of Rare Disorders (NORD) https://rarediseases.org	A 501(c)(3) organization, NORD is a patient advocacy organization dedicated to individuals with rare diseases and the organizations that serve them. NORD, along with its more than 260 patient organization members, is committed to the identification, treatment, and cure of rare disorders through programs of education, advocacy, research, and patient services
Maternal and Child Health Bureau (MCHB) within the Health Resources and Services Administration (HRSA) https://mchb.hrsa.gov/maternal-child-health-topics/children-and-youth-special-health-needs	MCHB provides leadership and resources to the nation in order to improve the quality of life for children with special health needs and their families. MCHB provides support for programs that support states, communities, and organizations to improve systems of care for all children with special health care needs
Coalition for Genetic Fairness www.geneticfairness.org/	Advocacy group for federal legislation regarding genetics discrimination through the National Partnership for Women & Families
The American Society of Human Genetics (ASHG) www.ashg.org	Part of the mission of ASHG is “to advance human genetics in science, health and society”. According to their mission statement, “this mission is only possible if the right policies are in place to support scientific discovery, the translation of scientific discoveries into health advances, and the appropriate application of genetics in society”. ASHG therefore analyzes emerging policy issues, comments on pending legislation and regulations, and advocates for evidence‐based policies that benefit science, personal and public health, and society at large

### 7.2 Genetic registries

Broadly speaking, genetic registries are databases of information collected on individuals with a specific inherited or genetic disease or condition. The data collected in registries will vary depending on the specific purpose(s) of the registry. Some registries exist to facilitate contact of patients with clinicians and are useful for organizing the disease community and clinical cohorts. Other registries exists to facilitate the advancement of research and include clinical and/or laboratory information. The resources listed below will provide a few widely used resources; however, they do not represent an exhaustive list (Table 2).

**Table 2:** Genetic databases. Listed are the patient registry databases and research registries and databases used for basic and clinical research

Registry name and website	Short description
Patient registries	
The National Institutes of Health *List of Registries* https://www.nih.gov/health-information/nih-clinical-research-trials-you/list-registries	Provides information about registry efforts at the national level and therefore do not include many local groups that can offer valuable assistance to individuals and their families
Registry of Patient Registries (RoPR), Agency for Healthcare Research and Quality (AHRQ), U.S. Department of Health and Human Services htttps://patientregistry.ahrq.gov/	RoPR is a database of registry specific information intended to promote collaboration, reduce redundancy and improve transparency
Research registries & databases	
National Center for Biotechnology Information (NCBI), National Library of Medicine https://www.ncbi.nlm.nih.gov/guide/all/	NCBI creates automated systems for storing and analyzing knowledge about molecular biology, biochemistry, and genetics; facilitating the use of such databases and software by the research and medical community; coordinating efforts to gather biotechnology information both nationally and internationally; and performing research into advanced methods of computer‐based information processing for analyzing the structure and function of biologically important molecules. NCBI supports numerous registries actively used by clinicians and researchers. We mention a few below
Genetic Testing Registry https://www.ncbi.nlm.nih.gov/gtr/	Provides a central location for voluntary submission of genetic test information by providers. The scope includes the test's purpose, methodology, validity, evidence of the test's usefulness, and laboratory contacts and credentials. The overarching goal of the GTR is to advance the public health and research into the genetic basis of health and disease
ClinVar https://www.ncbi.nlm.nih.gov/clinvar/	Freely accessible, public archive of reports of the relationships among human variations and phenotypes, with supporting evidence. ClinVar thus facilitates access to and communication about the relationships asserted between human variation and observed health status, and the history of that interpretation
Database of Genotypes and Phenotypes (dbGap) https://www.ncbi.nlm.nih.gov/gap	An archive and distribution center for the description and results of studies that investigate the interaction of genotype and phenotype. These studies include genome‐wide association (GWAS), medical next‐generation sequencing, molecular diagnostic assays, as well as association between genotype and nonclinical traits
Database of Short Genetic Variation (dbSNP) https://www.ncbi.nlm.nih.gov/snp	Includes single‐nucleotide variations, microsatellites, and small‐scale insertions and deletions. dbSNP contains population‐specific frequency and genotype data, experimental conditions, molecular context, and mapping information for both neutral variations and clinical mutations
GeneBank https://www.ncbi.nlm.nih.gov/genbank/	The NIH genetic sequence database is an annotated collection of all publicly available DNA sequences. GenBank is part of the International Nucleotide Sequence Database Collaboration
Gene https://www.ncbi.nlm.nih.gov/gene	A searchable database of genes, focusing on genomes that have been completely sequenced and that have an active research community to contribute gene‐specific data. Information includes nomenclature, chromosomal localization, gene products, and their attributes (e.g., protein interactions), associated markers, phenotypes, interactions, and links to citations, sequences, variation details, maps, expression reports, homologs, protein domain content, and external databases

### 7.3 Additional resources

1. Online Mendelian Inheritance in Man (OMIM) (https://www.omim.org/) A comprehensive, authoritative compendium of human genes and genetic phenotypes that is freely available and updated daily. The full‐text, referenced overviews in OMIM contain information on all known Mendelian disorders and over 15,000 genes. OMIM is intended for use primarily by physicians and other professionals concerned with genetic disorders, by genetics researchers, and by advanced students in science and medicine.
2. GeneReviews (https://www.ncbi.nlm.nih.gov/books/NBK1116/) An international point‐of‐care resource for busy clinicians, that provides clinically relevant and medically actionable information for inherited conditions in a standardized journal‐style format, covering diagnosis, management, and genetic counseling for patients and their families. Each chapter in GeneReviews is written by one or more experts on the specific condition or disease and goes through a rigorous editing and peer review process before being published online.
3. PubMed, National Library of Medicine (http://www.ncbi.nlm.nih.gov/PubMed/) Basic search engine for biomedical research, including research and commentary regarding clinical research ethics and regulations.
4. Center for Disease Control and Prevention, Public Health Genomics (https://www.cdc.gov/genomics/) Provides timely and credible information for the effective and responsible translation of genomics research into population health benefits.
5. U.S. Surgeon General's My Family Health Portrait (https://familyhistory.hhs.gov/FHH/) A web‐based tool to collect family health history information to share with doctors and family members.
6. ClinicalTrials.gov (https://clinicaltrials.gov) A resource provided by the National Library of Medicine, this is a database of privately and publicly funded clinical studies conducted around the world.
7. National Newborn Screening and Genetics Resource Center (http://genes-r-us.uthscsa.edu/) Provides information and resources in the area of newborn screening and genetics to benefit health professionals, the public health community, consumers, and government officials.
8. National Information Resource on Ethics & Human Genetics (http://bioethics.georgetown.edu/nirehg/) From Georgetown University and funded by the National Human Genome Research Institute, this resource supports information services on topics related to ethics and human genetics.

## 8 CONCLUDING REMARKS

We summarized the history and the current state of medical genetics and genomics in the United States. The information herein included is, however, subject to constant change, as the number of certified genetic providers, commercially available genetic tests, advocacy groups and registries, and online resources is in permanent flux. Thus, although the historical aspects of this two‐part review will remain unaltered, the rest of it will benefit from frequent updates. The reader can refer to the pertinent references, some of which are updated on a regular basis, in order to have the most up‐to‐date information available.

## CONFLICTS OF INTEREST

None declared.
